# Survival and Surrogate Biomarkers in Interventional Oncology Trials: Pitfalls, Challenges, and Future Directions

**DOI:** 10.1007/s00270-025-04281-7

**Published:** 2026-01-16

**Authors:** Ana P. Gonzalez, Adam Swersky, Riad Salem

**Affiliations:** Department of Radiology, Section of Interventional Radiology, Northwestern Feinberg School of Medicine, 676 N. St. Clair, Suite 800, Chicago, IL USA

**Keywords:** Interventional oncology, Clinical trials, Endpoints, Artificial intelligence, Statistics

## Abstract

**Introduction:**

Interventional oncology (IO) is a key component of multidisciplinary cancer care, delivering minimally invasive therapies with safety and efficacy comparable to or surpassing conventional approaches. As IO enters a transformative era, the field must continue demonstrating value within the greater world of oncology, particularly through the study and application of surrogate endpoints.

**Surrogate Endpoint Definitions and Validation Criteria:**

A central requirement for advancing IO is the rigorous evaluation of treatment outcomes through translational research. Surrogate endpoints, including imaging-based criteria and serum biomarkers, expedite therapeutic assessment, yet their validity as predictors of clinical benefit remains under scrutiny.

**Regulatory and Methodological Challenges:**

Experience from prior IO studies underscores the complex interplay between surrogate endpoints, overall survival, and quality of life metrics. Variable regulatory acceptance, incomplete validation, and inconsistencies in endpoint definition and reporting continue to challenge their adoption.

**Primary Liver Tumors:**

In hepatocellular carcinoma (HCC), surrogate endpoints have informed treatment evaluation; however, their predictive strength and reproducibility remain variable across studies.

**Secondary Liver Malignancies:**

Applications in metastatic liver disease similarly rely on imaging and serum-based surrogates, though performance and reliability remain heterogeneous.

**Limitations:**

Uncertainty persists regarding the ability of surrogate endpoints to reliably predict durable clinical outcomes, limiting their broader applicability.

**Future Directions:**

Advancing IO will require the integration of modern trial methodologies, synthetic control arms, radiomics, and artificial intelligence to strengthen surrogate endpoint validation and facilitate broader clinical and regulatory acceptance.

**Conclusions:**

By embracing its characteristically innovative spirit while maintaining a critical lens on data interpretation, the IO community can not only advance therapeutic development but also reinforce its indispensability in oncology. The beginning of a new quarter century brings a pivotal juncture, with an opportunity to reimagine IO’s trajectory bridging technical ingenuity with the nuanced demands of modern cancer care.

## Introduction

### Common IO Surrogate Endpoints: Brief Overview

Overall survival (OS) is considered the gold standard endpoint in oncology trials to assess treatment efficacy and is defined as the time from the start of treatment until death from any cause [1]. Since OS can require years to obtain, surrogate endpoints are utilized when it is impractical to wait for OS data. These surrogate endpoints often provide earlier indications of treatment efficacy and can include a range of objective clinical data not limited to imaging criteria and/or serum analyses. At minimum, a surrogate biomarker must be biologically plausible. Progression free survival (PFS) is one such surrogate endpoint defined as the time from the start of treatment until disease progression or death [2]. Time to progression (TTP) also evaluates disease progression, but does not include death as an event, and is a surrogate assessment of treatment efficacy. Objective response rate (ORR) represents the proportion of patients in a trial with complete or partial response based on imaging criteria such as the modified response evaluation criteria in solid tumors (mRECIST) [3]. Duration of response (DoR), or the time from initial response to disease progression or death, indicates the sustainability of the treatment effect. Quality of life (QOL) metrics quantify overall well-being and incorporate physical, emotional, and social contributors to health. Surrogate biomarkers solely detected by serum analysis are faster and typically more accessible than OS data or non-laboratory surrogate endpoints but often provide an indirect evaluation of treatment response (Table 1) [4].

**Table 1:** Comparative summary of serum biomarkers in interventional oncology

Biomarker	Associated malignancy	Clinical use	Validation status
AFP	Hepatocellular carcinoma (HCC)	Prognostic and treatment response monitoring	Partially validated (context-specific)
PIVKA-II (DCP)	Hepatocellular carcinoma (HCC)	Adjunct to AFP; treatment response	Limited validation
CA 19–9	Cholangiocarcinoma	Monitoring and trend assessment	Confounded by biliary obstruction
CEA	Colorectal liver metastases	Trend monitoring post-IO	Limited correlation with survival
CA 15–3	Breast cancer liver metastases	Exploratory use in treatment response	Not validated; inconsistent data

## Surrogate Endpoint Definitions and Validation Criteria

Various criteria for surrogate endpoints have been proposed over the past decades, reflecting evolving perspectives on their definition and role in clinical trials. In 1989 Prentice defined a surrogate endpoint as valid if the null hypothesis of no treatment effect on the surrogate is equivalent to the null hypothesis of no treatment effect on the true clinical endpoint. [5] While scientifically sound, this definition is impractical for most clinical trial designs due to its strict statistical equivalence conditions that are rarely achievable outside of highly controlled settings. [6] The NIH Biomarkers Working Group (2001) described surrogate endpoints as biomarkers expected to predict clinical benefit or harm based on epidemiologic, therapeutic, or pathophysiologic evidence. This definition implies a simplified, linear relationship between the surrogate and the clinical outcome, overlooking functional or intermediate measures [7]. Ciani et al. [8] expanded the concept further to include both biomarkers and intermediate outcomes as surrogates for endpoints like OS and QoL. Despite strong support, concerns were raised about the vague definition of “intermediate outcome.” These definitions highlight the ongoing tension between conceptual clarity, statistical validity, and practical feasibility in the definition, validation, and application of surrogate endpoints in interventional oncology.

## Regulatory and Methodological Challenges

Regulatory acceptance of surrogate endpoints varies depending on the approval pathway. The US Food and Drug Administration (FDA) distinguishes between “validated” surrogate endpoints used for traditional approval and “reasonably likely” surrogates permitted under the accelerated approval pathway. The FDA’s Surrogate Endpoint Table, updated semi-annually, provides developers with context-specific guidance on the acceptability of various surrogate measures [9]. However, reliance on incompletely validated surrogates—particularly in the context of accelerated approvals—has led to post-marketing trials in which survival benefits were either unconfirmed or contradicted [10]. As a result, health technology assessment (HTA) bodies increasingly emphasize the need for robust evidence linking surrogate endpoints with hard clinical outcomes, including OS and QoL.

Comprehensive surrogacy analysis requires data from randomized clinical trials, as correlations derived from observational studies do not account for confounding variables. Factors such as baseline health status can simultaneously influence treatment selection, the surrogate measure, and clinical outcomes, limiting the validity of observational data. Randomization mitigates these biases by ensuring balanced comparison groups to accurately assess whether a treatment’s effect on a surrogate endpoint reflects the true treatment effect on a clinical endpoint. Ideally, surrogate validation should be conducted with data from multiple randomized trials to support trial-level meta-analyses. Nevertheless, such comprehensive surrogacy analysis is frequently constrained by lack of trial availability due to limited patient cohorts or incomplete trial data [6]. In settings where only a single randomized controlled trial is available, the Prentice criteria continue to be an applicable framework in surrogacy assessment, with its first three criteria requiring treatment effect on both OS and the surrogate endpoint, as well as a strong association between the surrogate and OS. The last criterion requires that the treatment’s effect on OS is fully mediated through the surrogate [5].

While conceptually robust, all four criteria are rarely met in IO trials, with the fourth criterion being the most difficult to satisfy. This has led to more flexible causal-inference approaches for surrogate analysis such as principal-stratification and meta-analytical R^2^ frameworks. Principal stratification is a causal inference framework used to evaluate whether a treatment’s effect on a clinical outcome is mediated through a surrogate endpoint. It defines subgroups or principal strata based on individuals’ potential surrogate responses. For example, in patients undergoing locoregional therapy (LRT), with tumor response rate as the surrogate, patients can be conceptually grouped into those who would experience partial response regardless of treatment, those who would partially respond only if treated, and those who would not respond to LRT at all. Although these counterfactual outcomes cannot be directly observed, statistical models are used to estimate these latent strata. By comparing survival outcomes across these groups, principal stratification helps determine whether the observed survival benefit is truly mediated by the treatment’s effect on the surrogate, or whether other mechanisms are responsible. [11] The meta-analytic R^2^ approach evaluates surrogate validity at the trial level by quantifying how much of the variation in treatment effects on the clinical outcome is explained by corresponding effects on the surrogate across multiple randomized trials; an R^2^ value ≥ 0.85 is generally considered indicative of strong surrogacy. [12] This surrogate endpoint validation approach requires a sufficient number of well-conducted trials and assumes consistent treatment effects and relationships across studies.

**Fig. 1:**
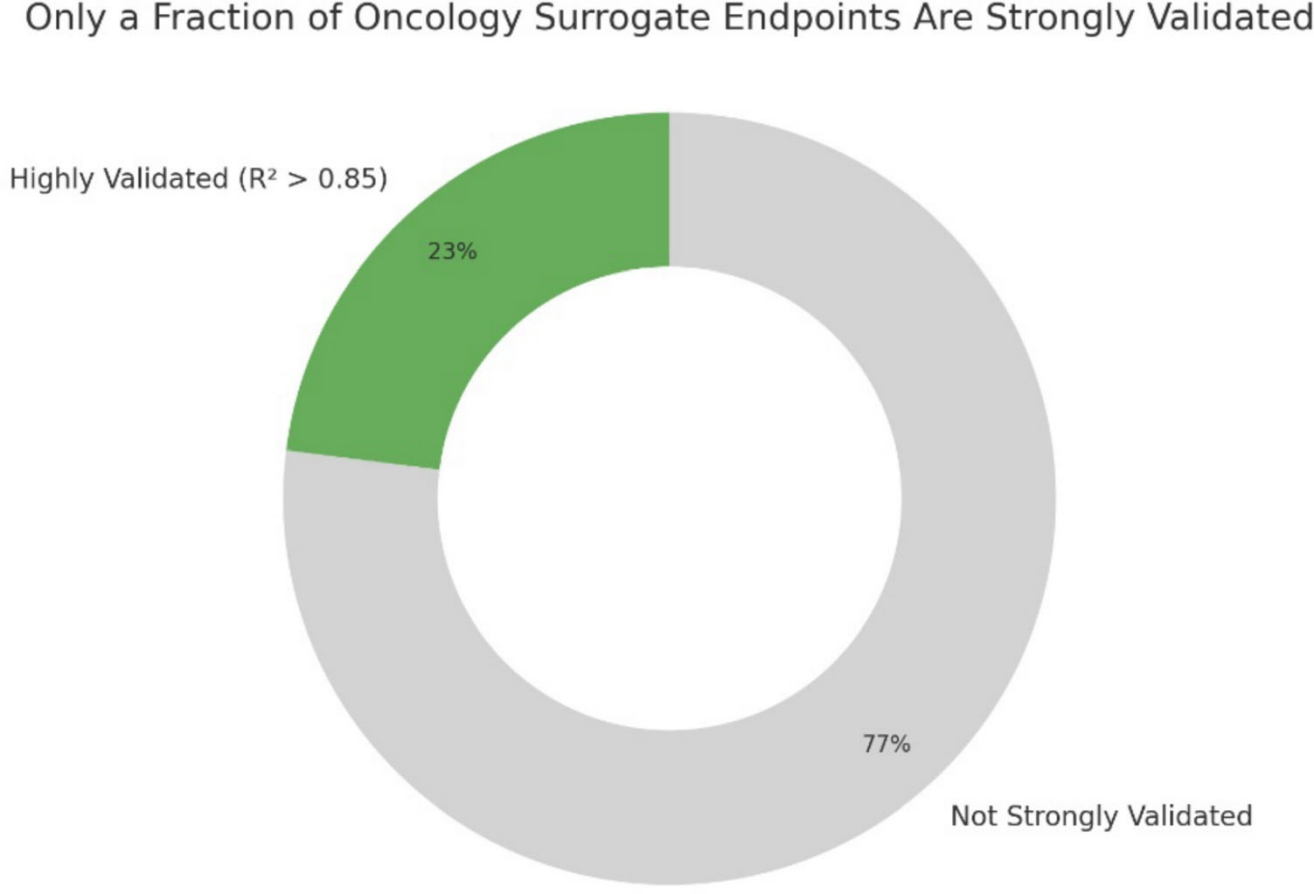
Fraction of surrogate endpoints validated in interventional oncology

The conceptual ambiguity, inconsistent definitions, and methodological challenges surrounding surrogate endpoints have historically led to inconsistent and often incomplete reporting in clinical trials. In response, standardized reporting guidelines, such as the Consolidated Standards of Reporting Trials (CONSORT) Surrogate extension, have developed to increase transparency of trials using surrogate endpoints. The CONSORT-Surrogate requires authors to state both the surrogate and its target outcome, justify its use, detail validation evidence, and report uncertainty metrics (e.g., trial-level R^2^) [13]. Despite growing recognition, an estimated 23% of cancer surrogate endpoints have achieved high trial-level correlation (R^2^ ≥ 0.85) with overall survival [10] (Fig. 1).

## Primary Liver Tumors

As of 2020, HCC was the second-leading cause of global cancer-related mortality [3, 14, 15]. Several treatment options exist for HCC in early and late-stage disease, including liver transplantation, surgical resection, systemic therapy, and thermal ablation. Transarterial LRTs for HCC include transarterial chemoembolization (TACE) and Yttrium-90 (Y-90) transarterial radioembolization (TARE). Over the past few decades, LRTs have been increasingly used for definitive therapy, finding niche benefit in selected populations by ‘bridging’ patients waiting on the organ recipient list, or by ‘downstaging’ patients who do not yet meet the Milan criteria for transplantation. In the relatively short history of IO, the study of HCC has been a central focus, with its diverse surrogate endpoints and biomarkers revealing the complexity of the narrative.

Alpha-fetoprotein (AFP), a glycoprotein produced by fetal liver cells and the yolk sac, is the most extensively studied biomarker for the screening, diagnosis, and monitoring of hepatocellular carcinoma (HCC), with decreasing levels indicating a favorable response to treatment [14]. AFP, however, lacks generalizability, as many patients are without elevated levels at baseline, making AFP ineligible for comparison with subsequent treatments. Des-gamma-carboxy prothrombin (DCP) is an abnormal prothrombin precursor for certain vitamin K-epoxide mutations that has been used as a biomarker for patients without elevated AFP. Biomarkers involving angiogenesis, such as hypoxia-inducible factor (HIF-alpha) and vascular endothelial growth factor (VEGF), have also offered prognostic value for LRTs. For example, a study in Italy found that responders to TACE had significantly lower levels of HIF-alpha compared to non-responders and a better prognosis with median OS of 28 months versus 17 months. [16] The same study also found significantly lower levels of vascular endothelial growth factor (VEGF) in TACE responders versus non-responders; however, unlike HIF-alpha, lower VEGF levels were not associated with improved overall survival [16].

Imaging biomarkers assess tumor size, viability, vascular invasion, and metastatic spread, serving as the foundation for the most reliable surrogate endpoints, particularly in the study of HCC, owing to its characteristic imaging features. In 1981, the World Health Organization (WHO) published the first solid tumor response criteria by assessing tumor size. In the mid-1990’s, the response evaluation criteria in solid tumors (RECIST) were published evaluating treatment response by assessing the longest diameter of a solid tumor target lesion. Methods based on size criteria (tumor shrinkage) have the strongest data supporting objective treatment responses. In 2009, mRECIST (modified RECIST) was published with specific adaptation to HCC, in that viable tumor burden was assessed by contrast-enhanced imaging. Since, mRECIST has been demonstrated to predict OS in patients following TACE. Other forms utilizing similar imaging assessments include the surrogate endpoints TTP and PFS. The PREMIERE trial (2016) was a prospective, open-label, single-center randomized controlled trial (RCT) designed to compare the efficacy of conventional TACE with glass Y-90 TARE in treating patients with unresectable HCC [17]. The primary endpoint of this trial was TTP, with OS, imaging response rates based on WHO and European Association for the Study of the Liver (EASL) criteria, and safety as secondary endpoints. This study found that the median TTP was significantly longer in the Y-90 TARE group compared to the TACE group (26 months vs. 6.8 months, *p* = 0.0012). [17] However, an improved TTP did not translate into improved OS. Both treatments were generally well-tolerated, with no significant differences in adverse events or objective response rates on imaging. This trial suggested that TARE may provide a more effective treatment option than TACE for patients with HCC, offering a longer duration of tumor control. Limitations of this trial included a small sample size, a single-center design, an open-label approach without blinding, and patient heterogeneity, including individuals with Barcelona Clinic Liver Cancer (BCLC) stages A and B of HCC, which may contribute to variability in outcomes due to differences in disease severity. While OS was used as a secondary endpoint, focusing primarily on TTP may not fully capture the overall benefit of treatment. The LEGACY (Local radioembolization using glass microspheres for the assessment of tumor control with Y-90) trial (2021) importantly assessed ORR and DoR in solitary unresectable HCC ≤ 8 cm. In this retrospective trial, patients underwent either lobar or selective hepatic radioembolization, achieving a best response or ORR of 88.3% according to localized mRECIST criteria. The DoR was ≥ 6 months, with a 3-year OS rate of 86.6% for all patients and 92.8% for neoadjuvant patients who subsequently underwent liver resection or transplantation. As with other retrospective trials, this trial lacked a control arm, but also offered a limited sample size and short follow-up median duration of 29.9 months without enough data to evaluate long-term outcomes such as OS and late adverse events [18]. These classic study critiques highlight the very importance of using surrogate biomarkers to understand our treatment effects from a common-sense perspective. While generating robust OS data is ideal, the individualized needs of our patients necessitate a more time-sensitive approach to achieving what truly matters to all of us—effective (and replicable) tumor eradication.

In intrahepatic cholangiocarcinoma, carbohydrate antigen 19–9 (CA 19–9) is commonly elevated and has been used to monitor therapeutic effect. CA 19–9, however, can be nonspecific and is affected by biliary obstruction, limiting its utility as a reliable surrogate markers. [19]

## Secondary Liver Malignancies

Research on non-HCC cancers treated with interventional oncology encounters similar limitations and challenges. Colorectal cancer (CRC) is the third most common malignancy worldwide, with approximately 30% of patients initially diagnosed with colorectal liver metastases (CLM) [20]. Gastrointestinal oncologists rely on biomarkers like carcinoembryonic antigen (CEA) to assess tumor burden, with declining levels serving as an indicator of a positive response to treatment. Additionally, clinicians analyze mutations such as KRAS, NRAS, and BRAF to inform and optimize targeted therapeutic strategies. Locoregional ablative therapies are recommended for those patients with small and few CLM lesions [21]. A combined analysis of three phase III trials (FOXFIRE, SIRFLOX, and FOXFIRE Global) (2017) demonstrated that selective internal radiation therapy (SIRT) with Y-90 resin microspheres, alongside FOLFOX chemotherapy, is a viable treatment option for CLM-predominant disease by significantly improving liver-specific PFS [22]. This trial, however, failed to show a significant improvement in overall PFS, and a subsequent study in 2017 similarly reported no notable enhancement in OS. The EPOCH trial (2021), an international open-label phase III RCT, demonstrated improved PFS and hepatic PFS with the addition of Y-90 glass microsphere TARE in CLM patients who progressed on first-line oxaliplatin- or irinotecan-based chemotherapy [23]. Although the trial showed improvements in PFS, it did not demonstrate a significant improvement in OS. Imaging biomarkers are increasingly utilized to evaluate responses to IO therapies, but their reliability across all situations remains challenging to establish. For instance, a retrospective trial evaluating the treatment of CLM with Y90-TARE revealed decreased OS in patients with primary right-sided CRC compared to left-sided CRC, despite no significant difference in imaging treatment response using RECIST criteria [21].

In patients with breast cancer liver metastases, CA 15–3 has been investigated as a marker of treatment response; however, evidence remains limited, and declines in CA 15–3 have not consistently correlated with improved survival or radiographic response [24].

## Other Applications

Other IO therapies are also poised to play an increasingly large role, such as image-guided percutaneous ablation (IGA) using radiofrequency, microwave, or cryoablation for treating small or unresectable renal cell carcinoma (RCC). However, most evidence supporting the non-inferiority of IGA to partial or radical nephrectomy stems from retrospective trials, which are often limited by issues such as data gathering and treatment allocation [25].

## Limitations and Future Directions

Surrogate biomarkers have been worth their weight in gold in advancing the field of IO forward. They must, however, continue to be met with skepticism at every level to ensure they are validated to predict the true clinical outcomes of interest, whether they be survival or quality-based. Validation is most securely achieved through large RCTs, which are unfortunately hard to come by in the world of IO. The small sample sizes in typical IO RCTs raise concerns regarding their statistical power to detect minimal differences, not unlike challenges faced in other areas of healthcare and oncology. Additionally, small sample sizes in RCTs restrict the generalizability of findings to diverse settings and populations. Surrogate biomarkers are unfortunately very context-dependent and may not capture all of the effects of an intervention.

Retrospective trials lack control arms, introduce the potential for selection bias, and compromise validity and reliability; the typical open-label designs in many IO studies introduce the possibility for observer bias in radiological assessments. These pitfalls underscore the necessity for systematic meta-analyses to integrate valid data and estimate treatment effects when individual randomized controlled trials are not sufficiently large enough to provide conclusive results [26]. Both the PREMIERE and LEGACY trials were limited by selective enrollment, lack of control arms, and reliance on surrogate outcomes without linked comparison to OS, whereas the randomized EPOCH trial demonstrated improved PFS without an OS benefit. The lack of demonstrated survival benefit may shroud other important conclusions about the overall benefit of these therapies. OS directly measures the length of time patients live after receiving treatment, regardless of the disease’s progression. Clinicians often rely on OS data to make informed treatment decisions, but it remains to be seen if this dogma translates to the most efficient progress in therapeutic development. That the FDA prefers OS as the primary endpoint for IO clinical trials is a significant hurdle in and of itself. Interventional and surgical cancer treatments require more concrete evidence of clinical benefit than pharmaceutical approvals due to the complexity and variability of procedural outcomes, as evidenced by half of the FDA cancer pharmaceutical approvals between 2009 and 2014 based off of studies reliant on surrogate endpoints as their primary endpoints [27].

Innovative synthetic trial designs may enhance patient accrual for clinical trials and streamline the evaluation of new treatments. Cancer registries are databases that collect, store, and manage cancer patient data at a population level, including demographics, diagnoses, treatments, outcomes, and long-term monitoring data [28, 29]. They facilitate clinical trial recruitment by helping identify eligible participants who meet the criteria and influence trial design with the incorporation of external control groups otherwise known as synthetic control arms (SCAs). SCAs can leverage external data (i.e., electronic health records) to establish virtual control groups, a particularly valuable approach for oncologic diseases with limited patient populations. While SCAs offer an innovative solution to trial recruitment and comparator limitations, they raise ethical and methodological concerns. Key issues include potential bias from unmeasured confounders, inconsistencies in data quality between real-world and trial settings, and the lack of informed consent for patients in external datasets. Stepped-wedge cluster randomized trial designs involve the sequential introduction of a particular intervention across different contexts, with each cluster serving as its own control before receiving the treatment [30].

Other promising advances in the assessment of cancer therapeutics include the use of gene expression and molecular biomarkers. Circulating tumor DNA (ctDNA) consists of fragments of DNA shed by tumor cells in the bloodstream, and monitoring ctDNA levels may provide insight into HCC tumor dynamics, treatment response, and the presence of minimal residual disease [31]. Radiomics is another encouraging field that involves the extraction of large amounts of quantitative features from medical images. These features, which include texture, shape, and intensity, are used to create a more detailed and precise understanding of disease characteristics. A retrospective study in China demonstrated that a modified volumetric and quantitative imaging response criteria (qEASL) using 3-D slicing software may allow for more accurate detection of TACE non-responders and predict OS in treatment-naive HCC patients when compared to mRECIST [32].

Finally, financial, statistical, and technological considerations will also undoubtedly shape the future IO landscape. As healthcare costs rise, payers increasingly scrutinize the cost-effectiveness analyses (CEAs) within their own systems. CEAs use surrogate biomarkers and endpoints to predict long-term outcomes, aiding decision-making throughout clinical trials. The *p* value, a fundamental concept in statistical hypothesis testing, has faced substantial debate in clinical trials due to concerns about its lack of replicability and the far-reaching implications of its dichotomous “significant/not significant” interpretation. In 2016, the American Statistical Association issued a statement that *p* values should not be the sole determinant of research conclusions [33]. Artificial intelligence (AI) can optimize trial efficiency by assisting researchers with literature review, data analysis, and by providing automated writing assistance. The authors express an optimistic belief in AI's immense potential to guide IO stakeholders toward a sustainably bright future.

## Conclusions

The relatively short history of interventional oncology (IO) has been one characterized by remarkable innovation and ever-growing recognition within the multidisciplinary oncology community. Yet, the responsibility lies with interventional oncologists to sustain this momentum by embracing the rigor of translational research and collaborative discourse. Surrogate endpoints and biomarkers, though invaluable in expediting therapeutic evaluation, must be wielded with caution and validated through robust, context-sensitive clinical trials. By leveraging advancements such as synthetic trial designs, radiomics, and AI-driven insights, the IO community can transcend the limitations of traditional methodologies faced by landmark IO trials and champion patient-centric progress. Ultimately, the endurance of IO at the forefront of cancer care will depend not only on the ingenuity of its interventions but also on its ability to harmonize evidence-based practices with fluency in the evolving language of oncology. With our patients’ wishes in clear view, failure will not be an option, and IO will remain essential in shaping the future of cancer treatment.
